# GM1 Ganglioside Modifies α-Synuclein Toxicity and is Neuroprotective in a Rat α-Synuclein Model of Parkinson’s Disease

**DOI:** 10.1038/s41598-019-42847-x

**Published:** 2019-06-10

**Authors:** Jay S. Schneider, Radha Aras, Courtney K. Williams, James B. Koprich, Jonathan M. Brotchie, Vikrant Singh

**Affiliations:** 1 Department of Pathology, Anatomy and Cell Biology, Thomas Jefferson University, Philadelphia, PA 19107 USA; 2 Toronto Western Research Institute, Toronto Western Hospital, University Health Network, Toronto, Ontario M5T 2S8 Canada

**Keywords:** Parkinson's disease, Cell death in the nervous system

## Abstract

While GM1 may interact with α-synuclein *in vitro* to inhibit aggregation, the ability of GM1 to protect against α-synuclein toxicity *in vivo* has not been investigated. We used targeted adeno-associated viral vector (AAV) overexpression of human mutant α-synuclein (A53T) in the rat substantia nigra (SN) to produce degeneration of SN dopamine neurons, loss of striatal dopamine levels, and behavioral impairment. Some animals received daily GM1 ganglioside administration for 6 weeks, beginning 24 hours after AAV-A53T administration or delayed start GM1 administration for 5 weeks beginning 3 weeks after AAV-A53T administration. Both types of GM1 administration protected against loss of SN dopamine neurons and striatal dopamine levels, reduced α-synuclein aggregation, and delayed start administration of GM1 reversed early appearing behavioral deficits. These results extend prior positive results in MPTP models, are consistent with the results of a small clinical study of GM1 in PD patients that showed slowing of symptom progression with chronic use, and argue for the continued refinement and development of GM1 as a potential disease modifying therapy for PD.

## Introduction

Parkinson’s disease (PD) is a neurodegenerative disorder characterized by loss of dopamine (DA)-producing neurons in the substantia nigra pars compacta (SNc), decreased levels of DA primarily in the caudate nucleus and putamen, accumulation of insoluble α-synuclein aggregates (i.e., Lewy bodies and Lewy neurites)^1^, and a slowly progressive worsening of clinical symptoms. Current pharmacotherapies for PD improve many of the motor signs and symptoms of the disease but no drug has yet been identified that definitively slows or stops the progression of PD. Disease modifying therapies that can alter clinical progression are sorely needed, however, efforts at finding such therapies have been limited in part due to uncertainty regarding the pathogenic processes contributing to DA neuron degeneration in PD that should be targeted by a disease modifying therapy.

Research from our group^2,3^ and others^4,5^ suggests that one potential pathogenic mechanism contributing to PD may involve dysregulation of ganglioside synthesis and expression that may contribute to the vulnerability and degeneration of DA neurons in PD. Gangliosides are glycosphingolipids bearing a ceramide anchor, an oligosaccharide, and one or more sialic acid residues^6^. The major ganglioside species in brain are GM1, GD1a, GD1b, and GT1b^7^, all contributing to the lipid composition of plasma and intracellular membranes. GM1 and other gangliosides are components of membrane signaling domains called lipid rafts, and in this regard, GM1 contributes to regulating signal transduction for directing neuronal development and cell survival and for modulating a wide variety of cell functions^8^. Further, at least four proteins associated with PD (LRRK2, Parkin, PINK1, and α-synuclein), have been found to associate with lipid rafts and some colocalize with GM1 (along with other raft markers) suggesting that alterations of the GM1-raft association could influence cellular functions dependent on these proteins^9,10^. In addition to effects exerted at the plasma membrane, GM1 also acts intracellularly where it influences Ca^2+^ homeostasis, mitochondrial function, and lysosomal integrity, among other processes critical for normal cell function and survival^11–14^. Preclinical studies have shown that treatment with GM1 can be neurotrophic or neuroprotective following different types of lesions [ex.,^15,16^] resulting in significant biochemical and behavioral recovery. In particular, GM1 rescued damaged SNc DA neurons, increased striatal DA levels and enhanced DA synthetic capacity in residual DA neurons in neurotoxin (MPTP)-induced models of PD^17–23^. Clinical studies of GM1 in PD patients have also provided evidence for slowing of symptom progression with GM1 use^24,25^. Despite the positive preclinical and clinical findings related to GM1 and PD, the mechanisms through which GM1 exerts its potential neuroprotective effects are still uncertain.

Alpha-synuclein fibrillation and aggregation are considered to be important contributors to PD pathophysiology^26^, with α-synuclein-containing cytoplasmic inclusions a histological hallmark of the disease^27^. Recent development of animal models of PD that reproduce this aspect of the disease have been achieved using viral vector delivery of α-synuclein directly to SNc DA neurons. In particular, several studies have demonstrated PD-relevant and progressive neuropathological (including development of insoluble α-synuclein aggregates) and behavioral features in mice^28^, rats^29,30^, and non-human primates^31^ following SNc-targeted AAV-driven overexpression of human mutant A53T α-synuclein. Binding of α-synuclein to GM1, *in vitro*, inhibits fibril formation, dependent upon the amount of GM1 present^8^, with a similar effect of GM1 on A53T mutant α-synuclein^8^. Additionally, the interaction of GM1 and α-synuclein *in vitro* led to a complete resistance to fibrillar aggregation of acetylated α-synuclein, raising the possibility that binding to GM1-rich membranes could protect monomeric α-synuclein from pathogenic aggregation^32^. Using the AAV-A53T α-synuclein rat model, the present study was conducted to investigate the extent to which GM1 ganglioside administration could protect against α-synuclein toxicity and development of PD-relevant pathological changes and behavioral deficits.

## Results

### Early start GM1 administration partially protects motor behavior and striatal DA levels

**Figure 1:**
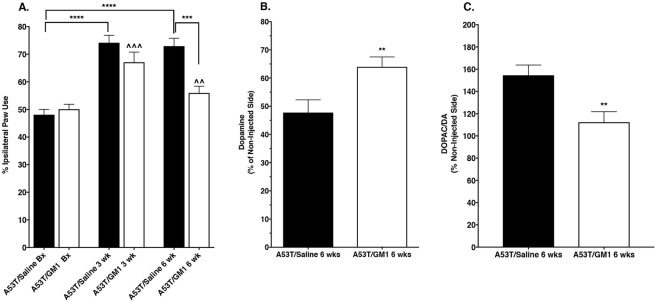
Protective effect of early start GM1on spontaneous forelimb use and striatal dopamine levels. (**A**) At 3 weeks following AAV-A53T α-synuclein injection, saline-treated animals (N = 15) favored the use of the limb ipsilateral to the injection and this response continued to be observed at 6 weeks post virus injection. In animals that received GM1 administration beginning 24 hours after AAV-A53T α-synuclein injection (N = 21), use of the limb ipsilateral to the injection was also favored at 3 weeks post injection, but with continued GM1 use, this response was reduced by 6 weeks post virus injection. ****P < 0.0001 vs. baseline (A53T/Saline Bx); ***P = 0.0005; ^^^P = 0.0003 vs. baseline (A53T/GM1 Bx); ^^P = 0.0055 vs. 3 wk. (**B**) Striatal DA levels were significantly higher in GM1-treated animals vs. saline-treated animals (**P = 0.0072). (**C**) Striatal DOPAC/DA ratios were significantly higher in saline-treated animals vs. GM1-treated animals (**P = 0.0040).

The cylinder test was used to assess spontaneous forelimb use in AAV-A53T α-synuclein-transduced animals and there was a significant main effect of treatment, with a protective effect observed in GM1-treated animals (F_(5,102)_ = 16.59, P < 0.0001). Animals that received AAV-A53T α-synuclein followed by saline for 6 weeks developed a significant asymmetry in paw use with preference for making contact with the cylinder with ipsilateral forepaw relative to the side of virus injection. At 3 weeks following AAV-A53T α-synuclein injection, saline-treated animals already showed a significant increase in percent ipsilateral limb use that continued to be observed at 6 weeks post virus injection (mean ± SEM: baseline: 48.1 ± 1.9%; 3 weeks: 74.2 ± 2.7%, 6 weeks: 72.9 ± 2.9%) (Fig. 1A). In animals that received GM1 administration beginning 24 hours after AAV-A53T α-synuclein injection, limb use asymmetry (percent ipsilateral limb use) was also increased at 3 weeks and 6 weeks after AAV-A53T α-synuclein injection but this increase was only significantly different from baseline at 3 weeks (mean ± SEM: baseline: 50.1 ± 1.8%; 3 weeks: 67.1 ± 3.7%, 6 weeks: 56.0 ± 2.5%) (Fig. 1A). The amount of limb use asymmetry was significantly less in GM1-treated animals at 6 weeks compared to saline-treated animals at 6 weeks (P < 0.0001) (Fig. 1A). Animals that received AAV empty vector injections had no significant changes in limb use (% ipsilateral limb use: baseline: 48.8 ± 2.3%; 3 weeks: 53.7 ± 4.2%; 6 weeks: 58.5 ± 4.1%; (F_(2,9)_ = 2.521, P = 0.1173).

Six weeks following AAV-A53T α-synuclein injection, striatal DA levels on the side ipsilateral to the injection in saline-treated animals were 47.8 ± 4.5% of the DA levels on the side contralateral to the injection (i.e., the non-injected side). In animals that began receiving GM1 administration 24 hours after surgery, striatal DA levels on the side ipsilateral to the injection were 64.0 ± 3.5% of the DA levels on the side contralateral to the injection (t_(34)_ = 2.858, P = 0.0072 vs. saline-treated) (Fig. 1B, Supplementary Table 1). DOPAC/DA ratios on the side ipsilateral to the injection in the saline-treated animals were 154.5 ± 9.2% of the contralateral (non-injected) side while in GM1-treated animals, DOPAC/DA ratios on the side ipsilateral to the injection were 112.3 ± 9.5% of the contralateral (non-injected) side (t_(34)_ = 3.092, P = 0.0040 vs. saline-treated) (Fig. 1C). Injection of the AAV empty vector had no significant effects on striatal DA levels (non-injected side: 10.57 ± 0.47 µg/g wet weight; injected side: 11.04 ± 0.69 µg/g wet weight, N = 9, P = 0.5775).

**Figure 2:**
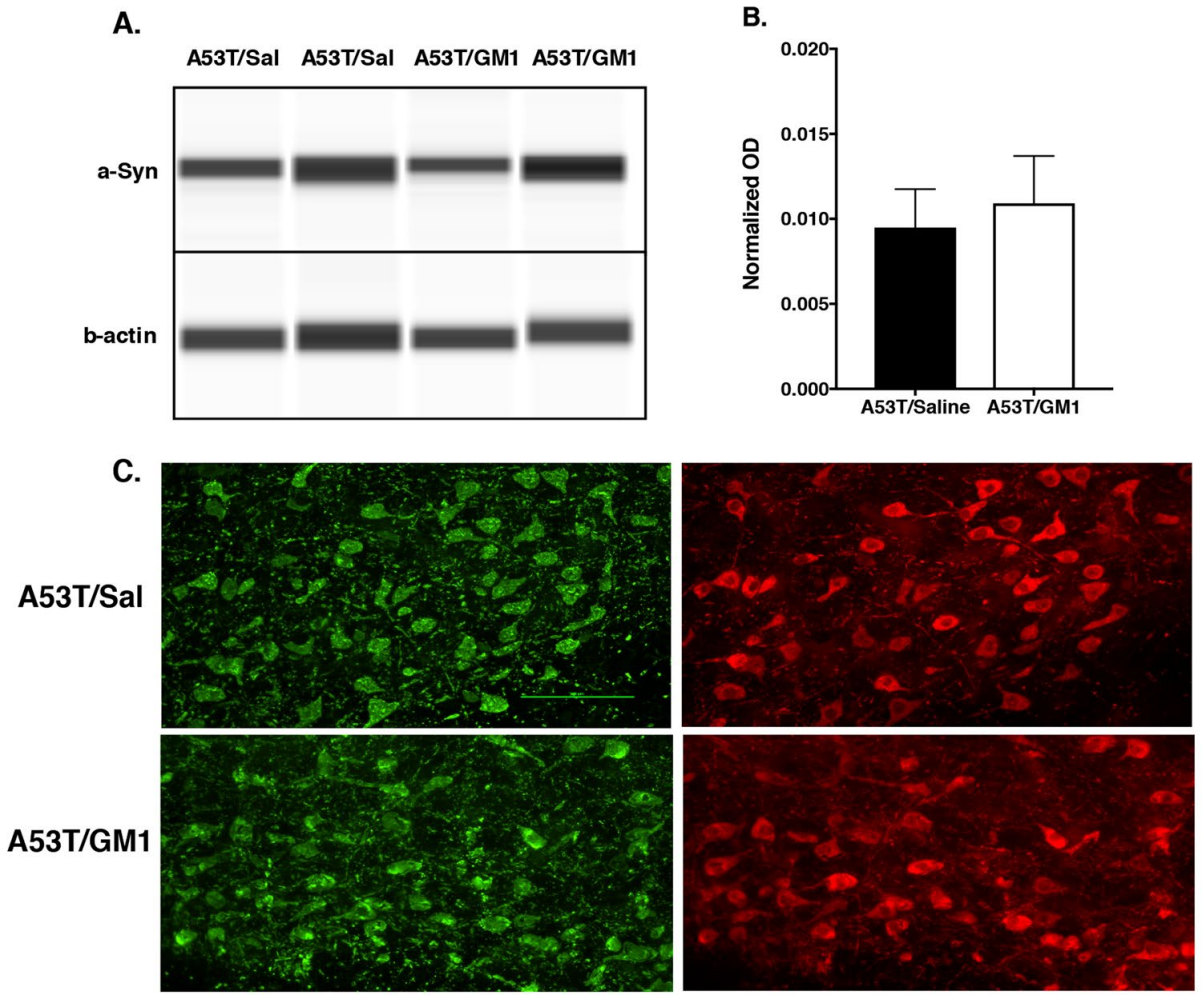
Early start GM1 administration did not affect α-synuclein expression or transport to the striatum. (**A**,**B**) When assessed 1 week following AAV-A53T α-synuclein injection, levels of striatal α-synuclein were no different in saline (N = 6) vs. GM1-treated animals (N = 6), suggesting no influence of GM1 on A53T α -synuclein transduction or transport to the striatum. Representative Wes Western blots are shown after cropping (full length images of blots are presented as Supplementary Fig. 2. (**C**) Double label immunofluorescence 1 week after AAV-A53T α -synuclein injection showed no differences between saline and GM1-treated animals in α -synuclein accumulation (green) in TH^+^ neurons (red) in the SNc.

The positive effects observed with GM1 administration beginning 24 hours after AAV-A53T α-synuclein injection cannot be explained by GM1 interfering with the transduction of the A53T α-synuclein gene by the AAV vector and expression of α-synuclein protein. When assessed at 1 week following AAV-A53T α-synuclein injection, levels of striatal α-synuclein were no different in saline vs. GM1-treated animals, suggesting no influence of GM1 on A53T α-synuclein transduction or transport to the striatum (normalized OD: 0.014 ± 0.004 and 0.017 ± 0.007, respectively, t_(12)_ = 0.4412, P = 0.6669) (Fig. 2A,B). Immunohistochemical evaluation of the SNc 1 week after AAV-A53T α-synuclein injection also showed no differences between saline and GM1-treated animals in α-synuclein accumulation in TH^+^ neurons (Fig. 2C,D).

### Early start GM1 administration partially protects SN neurons against α-synuclein-induced toxicity

**Figure 3:**
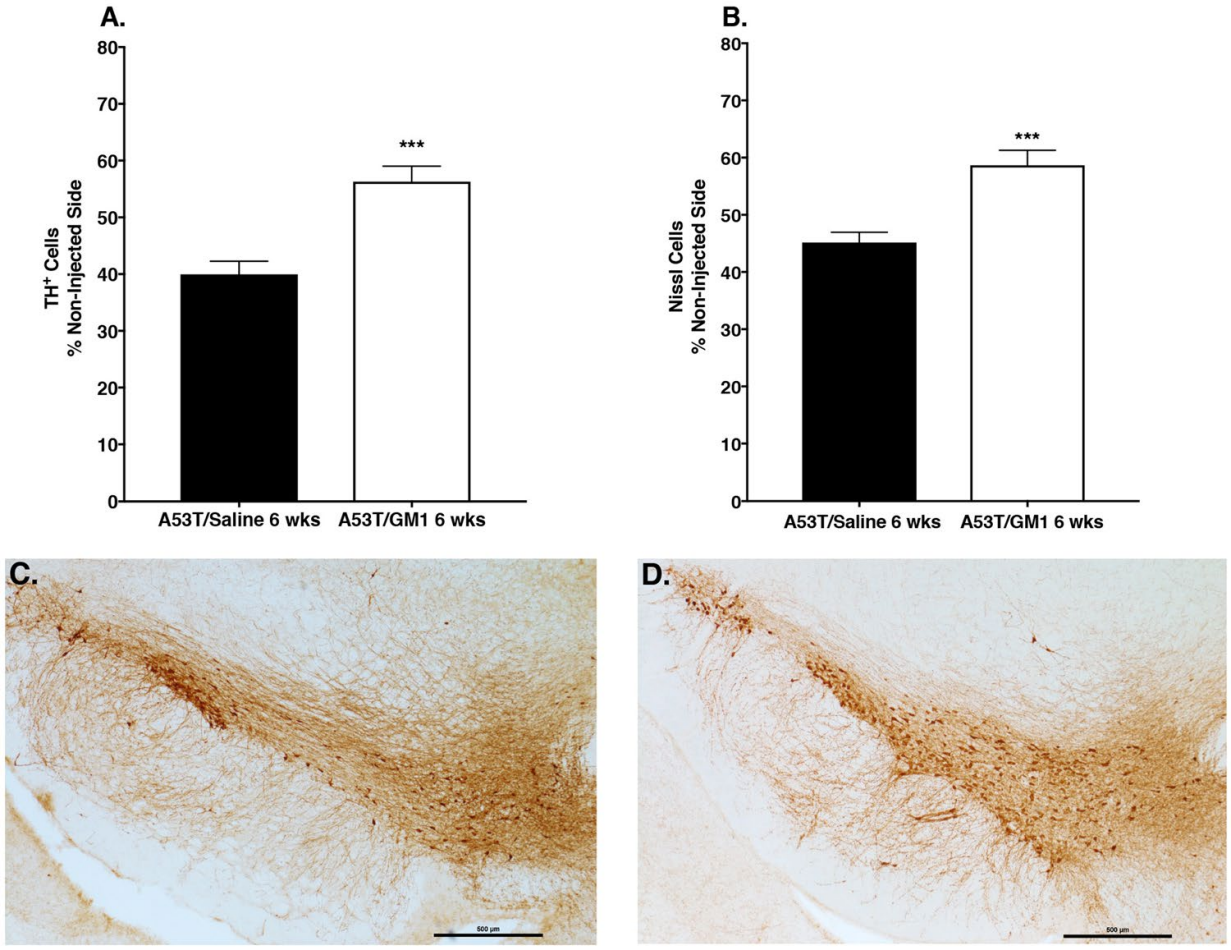
Early start GM1 administration partially protected against loss of SNc dopaminergic neurons. (**A**) There was a significant protective effect of early start GM1 administration (N = 17) on the number of TH^+^ cells (***P = 0.0002**)** and the number of Nissl-stained cells (**B**) (***P = 0.0004 vs. saline-treated) in the SNc, compared to saline-treated animals (N = 13). (**C**) Immunhistochemical staining of TH^+^ cells in the SNc showed significant cell loss in a saline-treated animal and (**D**), a partial sparing of TH^+^ cells in a GM1-treated animal.

To investigate the potential protective role of GM1 in the context of PD-relevant pathology, we examined the extent to which GM1 administration affected survival of A53T α-synuclein–overexpressing nigral DA neurons. The number of TH^+^ cells in the SNc was significantly decreased on the side ipsilateral to the injection: AAV-A53T-α-synuclein injection resulted in a 60.0 ± 2.3% loss of TH^+^ neurons, compared to the contralateral (non-injected) side (Fig. 3A,B). Animals that received early start GM1 administration had 43.7 ± 2.7% loss of TH^+^ neurons, compared to the non-injected side (t_(28)_ = 4.379, P = 0.0002) (Fig. 3A,B; Supplementary Table 2). Similarly, the number of cresyl violet-stained cells in the SNc was significantly influenced by AAV-A53T-α-synuclein injection and by GM1 treatment. AAV-A53T-α-synuclein injection caused a 54.9 ± 1.8% loss of cresyl violet-stained neurons, compared to the contralateral side. GM1-treated animals had only a 41.9 ± 2.5% loss of cresyl violet-stained neurons, compared to the contralateral side (t_(28)_ = 3.997, P = 0.0004) (Supplementary Table 2). Injection of the AAV empty vector had no significant effects on SN neurons: counts of TH^+^ neurons were 95.4 ± 1.1% of the non-injected side; counts of cresyl violet-stained neurons were 95.6 ± 1.3% of the non-injected side.

### Delayed start GM1 administration partially restores motor behavior and protects striatal DA levels

**Figure 4:**
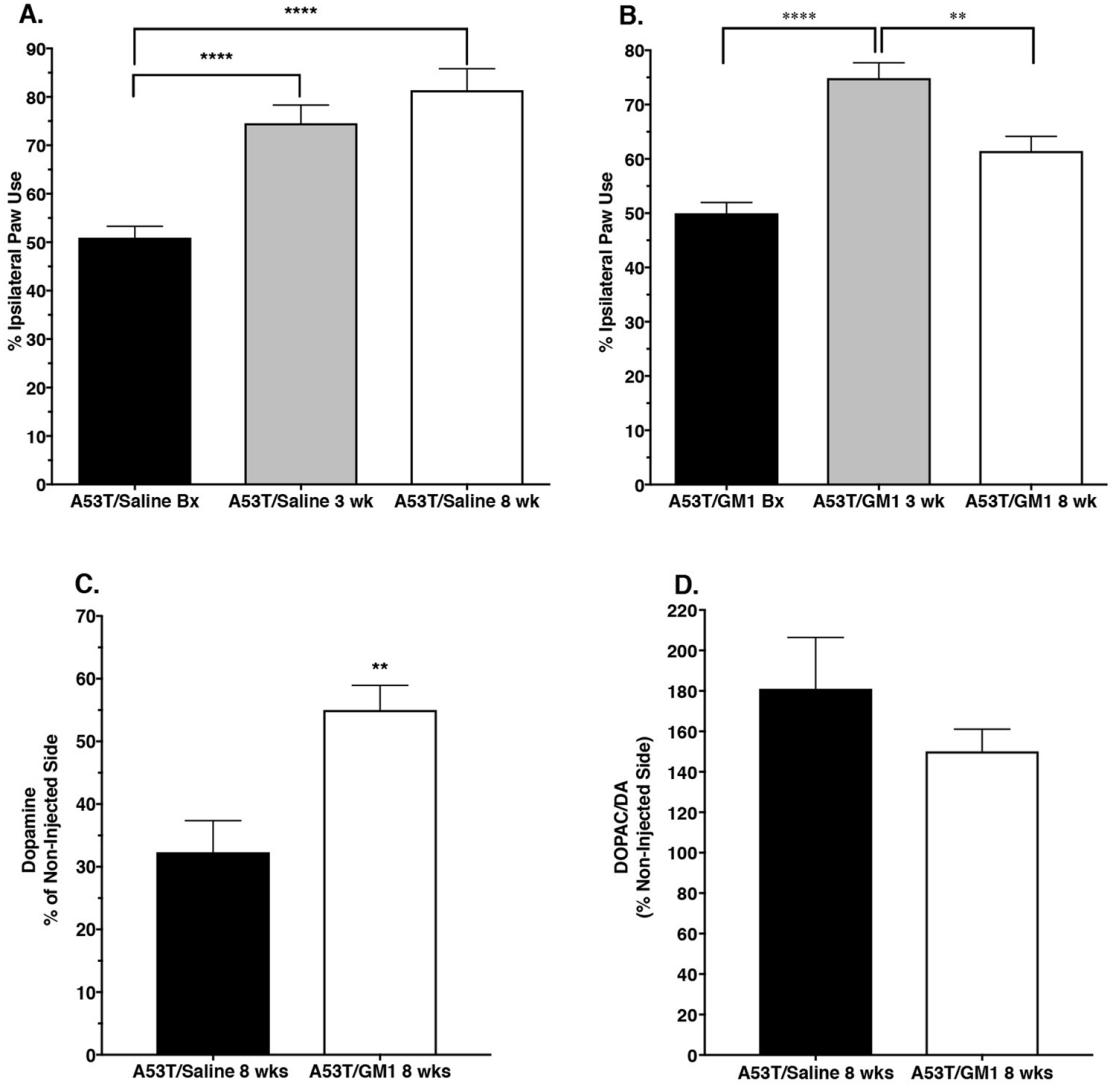
Delayed start GM1 administration partially restores motor function and partially protects striatal dopamine levels. (**A**) At 3 weeks following AAV-A53T α-synuclein injection, saline-treated animals (N = 11) showed a significant preference for using the limb ipsilateral to the injection and that bias continued to be observed at 8 weeks post virus injection (****P < 0.0001 vs. baseline (Bx). (**B**) In the delayed start GM1 group (N = 17), animals also showed a significant preference for using the limb ipsilateral to the injection at 3 weeks post virus injection (****P < 0.0001 vs. Bx) but after 5 weeks of GM1 use (starting immediately after 3 week testing), limb use asymmetry was significantly reduced (**P = 0.0075 vs. 3 weeks). (**C**) Striatal DA levels were significantly higher in GM1-treated animals (N = 17) vs. saline-treated animals (N = 11) (**P = 0.0013 vs. saline-treated). (**D**) DOPAC/DA ratios were lower in the GM1-treated (N = 17) but not significantly reduced compared to saline-treated animals (N = 11).

Animals that received AAV-A53T α-synuclein followed by saline for 8 weeks also developed a significant asymmetry in paw use with preference for making contact with the cylinder with the forepaw ipsilateral to the virus injection (Fig. 4A). When tested at 3 weeks following AAV-A53T α-synuclein injection, saline-treated animals showed a significant increase in percent ipsilateral limb use, similar to what was seen in the animals described earlier, that continued to be observed at 8 weeks post virus injection (mean ± SEM; baseline: 50.9 ± 2.4%; 3 weeks: 74.5 ± 3.8%, 8 weeks: 81.4 ± 4.5%) (Fig. 4A). There was a significant treatment effect favoring the GM1-treated group (F_(5,84)_ = 19.14, P < 0.0001). Animals assigned to the GM1 group had baseline percent ipsilateral limb use (50.0 ± 2.0%) comparable to that of the saline group and an increase in percent ipsilateral forelimb use at week 3 (74.9 ± 2.8%), also comparable to that observed in the saline group. These animals then received daily GM1 administration for the next five weeks and when tested again at week 8, showed a significant decrease in percent ipsilateral forelimb use (i.e, an increase in contralateral limb use) (61.5 ± 2.7%) compared to performance at week 3 just prior to receiving GM1 treatment (P = 0.0075 vs. week 3) (Fig. 4B).

Eight weeks following AAV-A53T α-synuclein injection, striatal DA levels on the virus injected side of the brain in saline-treated animals were 32.3 ± 5.0% of the DA levels on the contralateral (non-injected) side. In animals that began receiving GM1 administration 3 weeks after surgery, striatal DA levels on the virus ipsilateral (injected) side of the brain were 55.0 ± 3.9% of the DA levels on the contralateral side (t_(29)_ = 3.565, P = 0.0013 vs. saline-treated) (Fig. 4C). DOPAC levels on the injected side in the saline-treated animals were 69.4 ± 7.4% of the contralateral side while in GM1 treated animals, DOPAC levels on the injected side were 82.0 ± 8.4% of the non-injected side, an increase but not statistically significantly different from saline-treated animals. DOPAC/DA ratios on the side ipsilateral to the injection in the saline-treated animals were 181.1 ± 25.4% of the contralateral side while in GM1-treated animals, DOPAC/DA ratios on the side ipsilateral to the injection were 150.0 ± 10.9% of the contralateral side, although this difference was not statistically significant (Fig. 4D).

### Delayed start GM1 administration partially protects SN neurons against α-synuclein-induced toxicity

**Figure 5:**
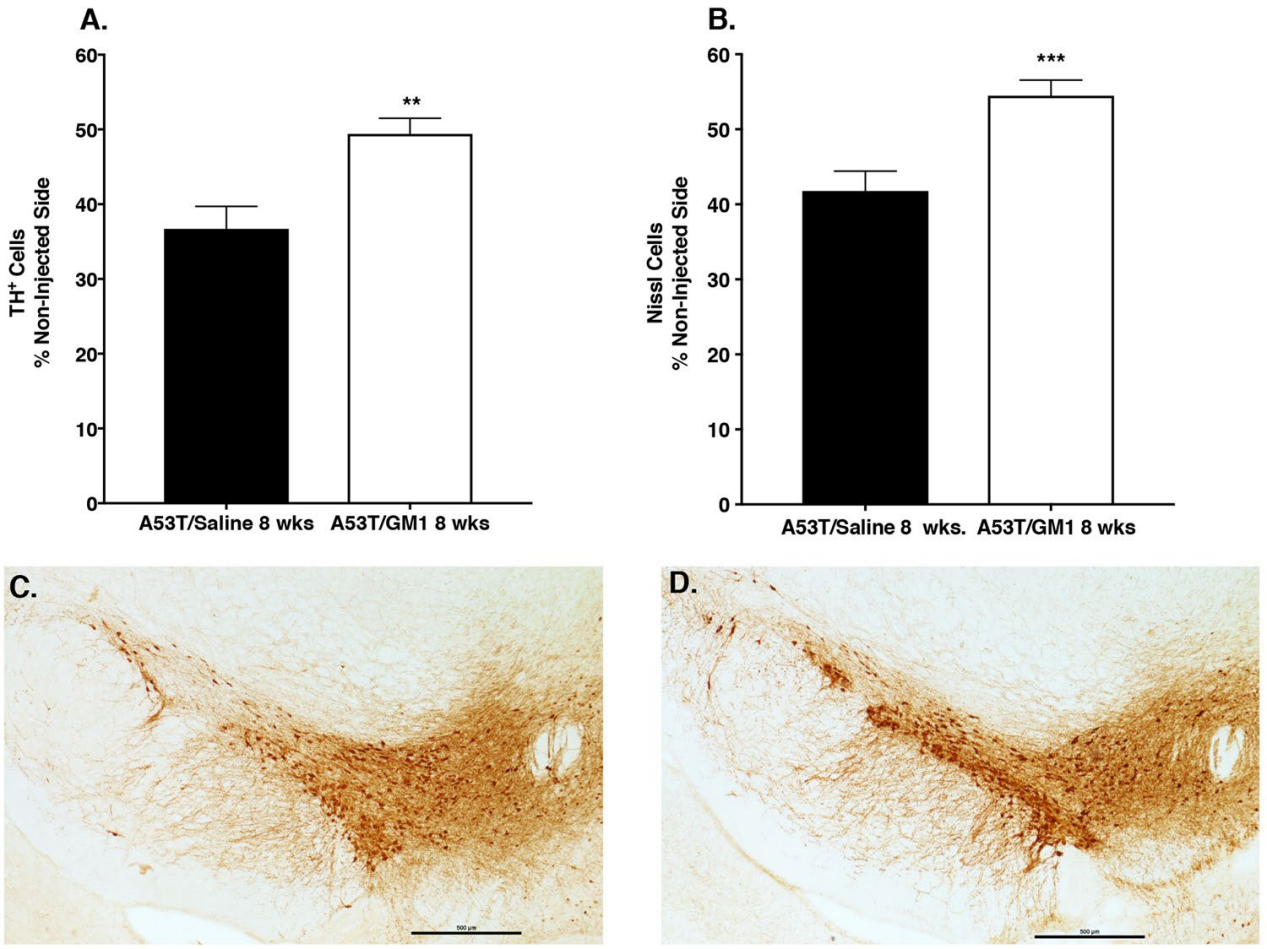
Delayed start GM1 administration partially protected against loss of SNc dopaminergic neurons. **(A)** There was a significant protective effect of delayed start GM1 administration (N = 17) on the number of TH^+^ cells (**P = 0.0013**)** and the number of Nissl-stained cells (**B**) (***P = 0.0008 vs. saline-treated) in the SNc, compared to saline-treated animals (N = 12). (**C**) Immunohistochemical staining of TH^+^ cells in the SNc showed significant cell loss in a saline-treated animal and (**D**), a partial paring of TH^+^ cells in a GM1-treated animal.

We examined the extent to which delayed start administration of GM1 could affect survival of A53T α-synuclein–overexpressing nigral DA neurons. The number of TH^+^ cells in the SNc was significantly decreased on the side ipsilateral to the injection: AAV-A53T-α-synuclein injection resulted in a 63.3 ± 3.0% loss of TH^+^ neurons, compared to the contralateral (non-injected) side. Animals that received delayed start GM1 administration had 50.6 ± 2.1% loss of TH^+^ neurons, compared to the contralateral side (t_(27)_ = 3.58, P = 0.0013) (Fig. 5A,B; Supplementary Table 2). The number of cresyl violet-stained cells in the SNc was also significantly influenced by AAV-A53T-α-synuclein injection and by delayed start GM1 treatment. AAV-A53T-α-synuclein injection caused a 58.2 ± 2.6% loss of cresyl violet-stained neurons, compared to the contralateral side. Delayed start GM1-treated animals had only a 45.5 ± 2.1% loss of cresyl violet-stained neurons, compared to the contralateral side (t_(27)_ = 3.796, P = 0.0008) (Supplementary Table 2).

### α-synuclein aggregation is reduced by GM1 administration

**Figure 6:**
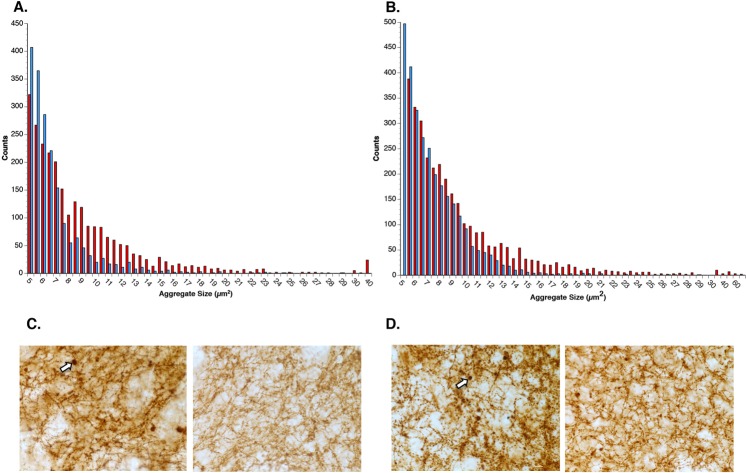
GM1 treatment reduces the size of α-synuclein-positive aggregates in the striatum. (**A**) The size distributions of striatal α-synuclein-positive aggregates in saline-treated (N = 6) (red bars) compared to early start GM1-treated (blue bars) animals (N = 6) were significantly different (Kolmogorov-Smirnov D = 0.2945, P < 0.0001), with a clear shift to larger numbers of smaller sized aggregates and fewer larger sized aggregates in the early GM1 group compared to the saline-treated group. (**B**) The size distributions of striatal α-synuclein-positive aggregates in the delayed GM1-treated group (N = 8) (blue bars) was also significantly different (Kolmogorov-Smirnov D = 0.154, P < 0.0001) than size distribution of aggregates in the saline-treated group (N = 7) (red bars), with greater numbers of larger sized aggregates in the saline-treated group compared to the GM1 group. (**C**) Photomicrographs of α-synuclein immunohistochemical staining in the striatum of saline-treated (left) and early start GM1-treated (right) animals. Sizes of aggregates are notably smaller in the GM1-treated animal. Arrow points to large α-synuclein-positive aggregate. (**D**) Photomicrographs of α-synuclein immunohistochemical staining in the striatum of saline-treated (left) and delayed start GM1-treated (right) animals. Sizes of aggregates are smaller in the delayed start GM1-treated animal, but the effect was not as dramatic as in the early-start GM1 animals. Arrow points to large α-synuclein-positive aggregate.

Since previous *in vitro* studies suggest that GM1 and α-synuclein (both wildtype and the A53T mutation) interact in a way that potentially inhibits pathogenic aggregation^8^, we hypothesized that GM1 administration to AAV-A53T overexpressing animals would result in less α-synuclein aggregation and thus smaller sized aggregates. Thus, we examined the effect of early or delayed start GM1 administration on the size of α-synuclein-positive swellings/aggregates in the striatum. The size distributions of striatal α-synuclein-positive aggregates in saline-treated compared to GM1-treated (starting 24 hours after AAV-A53T administration) were significantly different (Kolmogorov-Smirnov D = 0.2945, P < 0.0001), with a clear shift to larger numbers of smaller sized aggregates and fewer larger sized aggregates in the GM1 group compared to the saline group (Fig. 6A,C). The maximum aggregate size measured in the saline group was 52.5 µm^2^ while the maximum aggregate size measured in the GM1-treated animals was 28.8 µm^2^.

Similar results were obtained with measurements of aggregate size distributions in animals with delayed start GM1 administration compared to saline-treated animals. The size distributions of striatal α-synuclein-positive aggregates in saline-treated compared to GM1-treated (starting 3 weeks after AAV-A53T administration) were significantly different (Kolmogorov-Smirnov D = 0.154, P < 0.0001), with greater numbers of larger sized aggregates in the saline group compared to the GM1 group (Fig. 6B,D). The maximum aggregate size measured in the saline group was 66.9 µm^2^ while the maximum aggregate size measured in the GM1-treated animals was 29.3 µm^2^. Compared to animals that started receiving GM1 24 hours after AAV-A53T administration, animals that received GM1 starting 3 weeks after AAV-A53T administration had greater numbers of intermediate-sized aggregates (approximately 7–12 µm^2^), but like the early start GM1 group, had fewer larger sized aggregates than the saline group.

Although we did not quantify the accumulation of Ser129-phosphorylated α-synuclein, immunohistochemical visualization of Ser129-phosphorylated α-synuclein-positive neurons and neurites in the SN of GM1-treated and saline-treated animals showed less intense cytoplasmic Ser129-phosphorylated α-synuclein staining and fewer/smaller nuclear aggregates in GM1-treated animals compared to saline-treated animals (Supplementary Fig. 1).

## Discussion

GM1 ganglioside has had beneficial effects on striatal DA levels and behavior and has protected SNc DA neurons against degeneration in neurotoxin (primarily MPTP) models of PD^17–23^. GM1 has also been shown to have symptomatic effects and with extended use, may slow symptom progression in PD patients^24,33^. The present study demonstrates that GM1 administration is also able to exert neuroprotective and potentially neurorestorative effects on the nigrostriatal DA system in a PD model characterized by targeted overexpression of human mutated α-synuclein (A53T) in SNc neurons^29^. These data are particularly interesting considering that other compounds that have been found to be neuroprotective in MPTP mouse or nonhuman primate models of PD have not had this preclinical success translated to the α-synuclein model or to the clinic. Although there are several possible reasons why compounds such as GDNF, neurturin, CoQ10, CEP-1347, GPI-1485, pioglitazone, exenatide, and others that showed promising neuroprotective effects in MPTP models failed to demonstrate clear disease-modifying effects in clinical trials^34^, the relevance and translatability of the MPTP (and other toxin-based) models of PD have been called into question^35^, based on the assumption that the toxin models do not faithfully replicate key aspects of the disease^35^. However, clinical translational failures may be as much due to specific characteristics of the compounds tested as to deficiencies in the preclinical models. Here we show that unlike these other compounds, GM1 is neuroprotective in mouse and nonhuman primate MPTP models^17,18,21,23^, is neuroprotective in an AAV-α-synuclein model that reproduces DA neuron degeneration and dysfunction based on a core molecular feature of PD (i.e., toxicity associated with accumulation of aberrant α-synuclein^29)^, and that these preclinical successes have translated to positive effects in initial clinical trials^24,33^.

In the current study, a behavioral deficit was observed on the cylinder test at 3 weeks following AAV-A53T α-synuclein injection. Although we did not euthanize animals in this study at this 3 week period, a previous in-depth characterization of this model, using exactly the same vector and vector concentration as used here, showed that the cylinder test deficit observed at 3 weeks appeared in the absence of significant loss of SN DA neurons or striatal DA levels but in the presence of moderate dystrophic axonal morphology in the striatum^29^. Animals treated with GM1 beginning 24 hours after AAV-A53T α-synuclein injection were already beginning to show reduced use of the forelimb contralateral to the injection when tested at 3 weeks and by 6 weeks following AAV-A53T α-synuclein injection, this effect became statistically significant, with the ratio of ipsilateral/contralateral forelimb use approximating that observed at baseline (prior to AAV-A53T α-synuclein injection). At 6 weeks, GM1-treated animals also had significantly higher striatal DA levels than saline-treated animals. We also showed that early start GM1 treatment did not interfere with the expression or transport of α-synuclein. These results suggest that early start GM1 therapy, while not interfering with the transduction of A53T α-synuclein by the AAV vector and expression/transport of α-synuclein, did interfere with the pathological processes set in motion from the accumulation of α-synuclein.

Of considerable interest was the finding that delayed start GM1 treatment could significantly reverse the behavioral deficit observed at 3 weeks following AAV-A53T α-synuclein injection. As suggested by others^29^, the behavioral deficits seen at the 3 week time-point are a result of A53T α-synuclein-induced dysfunction of a relatively anatomically intact nigrostriatal pathway. Starting GM1 therapy at this time-point was able to afford some protection against the degeneration of SNc DA neurons and loss of striatal DA even once the pathological process of α-synuclein accumulation had begun, and was able to reverse the forelimb use deficit observed at 3 weeks, as observed at 8 weeks post AAV-A53T α-synuclein injection.

The mechanisms underlying the neuroprotective efficacy of GM1 in this model are not entirely known at this time. We observed that in animals administered GM1 there was decreased aggregation of α-synuclein and we have preliminary observations that GM1 may also decrease α-synuclein phosphorylation, potentially decreasing the accumulation of toxic forms of α-synuclein. Phosphorylation of α-synuclein at Ser129 promotes α-synuclein fibril formation^36^ and the majority of α-synuclein deposited in Lewy bodies in the PD brain is extensively phosphorylated at Ser129^36,37^. A53T α-synuclein as well as wild type α-synuclein can form insoluble toxic fibrillar aggregates and mutant α-synuclein may be more prone to aggregation and toxicity than wild type α-synuclein^38,39^. We found that both early start and delayed start use of GM1 following AAV-A53T α-synuclein injection reduced the size of α-synuclein aggregates measured in the striatum. *In vitro* studies have shown a direct association between GM1 and α-synuclein, attributed to interaction between helical α-synuclein and both the sialic acid and carbohydrate moieties of GM1, and that this association with GM1 inhibited fibrillation^8^. As we and others have shown decreased expression of GM1 and other complex gangliosides in the SN in PD^3,4^ and decreased expression of genes involved in ganglioside biosynthesis in DAergic neurons in the PD SN^2^, it is conceivable that reduced levels of gangliosides, and particularly GM1, in the PD SN may promote the accumulation of toxic α-synuclein and that administration of GM1 may provide sufficient amounts of this important sphingolipid so as to at least partially inhibit the toxic aggregation of α-synuclein and provide some level of protection to SN DA neurons. This possibility is supported by studies with *B4galnt1* knockout mice, devoid of GM1 and other a-series gangliosides, that reported increased amounts of α-synuclein aggregation in knockout mice devoid of GM1 that could at least be partially reduced by administration of GM1 and the semi-synthetic GM1 derivative LIGA-20^4^.

Another possible mechanism underlying the neuroprotective effects of GM1 observed in the current study may involve influences of GM1 on autophagy and lysosomal function. Dysfunction of the autophagy-lysosomal pathway has been suggested to contribute to PD pathology^40^. Overexpression of A53T-α-synuclein suppresses autophagy and pathogenic A53T-α-synuclein is poorly processed and cleared by chaperone-mediated autophagy^41,42^. Under conditions of impaired autophagy, GM1, either *in vivo* or *in vitro*, increased expression of autophagic markers and enhanced autophagy^43^. Depletion of endogenous gangliosides resulted in compromised lysosomal functions and suppression of autophagy, leading to accumulation of both α-synuclein and P123H β-synuclein in a cellular model of Lewy body disease^14^. It is possible that in the current study, autophagic processes were impaired by A53T-α-synuclein overexpression contributing to enhanced α-synuclein aggregation and DAergic cell loss and that GM1 treatment enhanced lysosomal function and increased autophagic clearance of α-synuclein. Additional studies are now needed to investigate this potential mechanism in regard to the neuroprotective effects of GM1 in this model.

The AAV-A53T α-synuclein model that we used for this study was the same model described previously as producing a specific and progressive degeneration of the nigrostriatal DA system^29^. A limitation of our study is that animals were not euthanized at early time-points following AAV-A53T α-synuclein injection and thus, the progressive nature of the pathology, as previously described, was not verified. However, in early pilot studies when establishing the model, we were able to detect a steady increase in forelimb use asymmetry over the first 3 weeks post-injection. Although we performed a limited examination of phosphorylated Ser 129 α-synuclein expression by immunohistochemistry, there were not sufficient tissues available for systematic evaluation of levels of phosphorylated Ser 129 α-synuclein. Likewise, the presence of insoluble phosphorylated α-synuclein aggregates was not directly assessed. Striatal α-synuclein aggregates have been shown to be extensively phosphorylated at Ser129 in rats with AAV-mediated A53T-α-synuclein overexpression^44^, however, in the current study, we only assessed total α-synuclein positive aggregates in the striatum and did not separately analyze Ser129-phosphorylated α-synuclein-positive aggregates. Follow-up studies are now needed to examine in more detail the relationship between GM1 administration and α-synuclein phosphorylation. Also, only one dose level of GM1 was used in this study and although the dose used was the same that has been shown to be neuroprotective in MPTP mouse models of PD, it is possible that the dose used may not have been optimal in the rat AAV-A53T α-synuclein model. Additionally, the effects of different durations of treatment with GM1 need to be investigated.

In conclusion, this study showed that GM1 ganglioside, previously shown to be neuroprotective in MPTP models of PD, also has neuroprotective effects in an AAV-A53T α-synuclein overexpression model of PD. Neuroprotective effects in this model, which is presumed to be more pathologically relevant to PD than neurotoxin models, is in agreement with our clinical experience with GM1 in PD patients, where prolonged use of GM1 produced evidence of a potential disease modifying effect. Development of treatments that directly impact the underlying disease processes in PD and that can slow neuronal cell death and symptom progression remain an unmet need of the PD population^45^. Based on previous work and the current results, GM1 continues to have potential to be such a treatment, with the potential to protect DA neurons from dying as well as rescue and restore function to damaged but viable neurons, and thus continued clinical development of GM1 for PD is indicated.

## Materials and Methods

### Vector

The vector used in this study was the generous gift of Drs. J. Koprich and J. Brotchie, Toronto Western Research Institute. Full details of vector design can be found in Koprich *et al*.^30^. Briefly, a chimeric adeno-associated vector (AAV) of a 1/2 serotype (capsid expresses AAV1 and AAV2 serotype proteins in a 1:1 ratio) with human A53T alpha synuclein expression driven by a chicken beta actin (CBA) promoter hybridized with the cytomegalovirus (CMV) immediate early enhancer sequence was used. A woodchuck post-transcriptional regulatory element (WPRE) and a bovine growth hormone polyadenylation sequence (bGH-polyA) were also incorporated to further enhance transcription following transduction. The vectors (AAV1/2-A53T and empty vector control) were produced by GeneDetect Ltd., Auckland, New Zealand. Viral titers were determined by quantitative PCR (Applied Biosystems 7900 QPCR) with primers directed to the WPRE region, thus representing the number of functional physical particles of AAV in the solution containing the genome to be delivered.

### Animals and vector delivery

All animal procedures were approved by the Thomas Jefferson University Institutional Animal Care and Use Committee and conducted in accordance with the National Institutes of Health Guide for the Care and Use of Laboratory Animals. Adult male Sprague-Dawley rats (Envigo), 250 to 300 g at the time of surgery, were housed three per cage with ad libitum access to food and water during a 12-hour light/dark cycle.

All surgeries were performed under general anesthesia using a mixture of ketamine (100 mg/kg, i.p.) and xylazine (10 mg/kg, i.p.). Once animals were anesthetized, the head was shaved and surgically prepped with Betadine solution and alcohol before being placed in a Kopf stereotaxic frame with the incisor bar set at approximately 3.5 mm below horizontal zero to achieve a flat skull position. The skin on the top of the skull was cut along the midline and retracted. Using coordinates AP: −5.3, L:2.2, D:7.5 below skull^46^, derived from bregma, a small burr hole was created over the SN on one side and a 36 gauge needle attached to a Hamilton syringe loaded with AAV-A53T-synuclein or empty vector control virus was slowly lowered into the brain and 2.0 µl was injected above the SN at a rate of 0.2 µl/min using a motorized syringe pump. Following a 5 minute wait period after completion of the injection, the needle was slowly withdrawn, the skin wound was closed, and post-surgery analgesia (meloxicam 1 mg/kg) was administered.

Some rats were randomly assigned to receive daily GM1 ganglioside (porcine brain derived GM1, 30 mg/kg, i.p., Qilu Pharmaceutical Co., Ltd.) or similar volume saline injections beginning 24 hours after AAV-A53T-synuclein surgery and lasting for 6 weeks (early start group). Other animals were randomly assigned to receive daily GM1 ganglioside (porcine brain derived GM1, 30 mg/kg, i.p., Qilu Pharmaceutical Co.) or similar volume saline injections beginning 3 weeks after AAV-A53T-synuclein surgery and lasting for 5 weeks (delayed start group). The dose of 30 mg/kg was selected based on the following information: this dose of GM1 was previously found to be effective in MPTP lesion models in mice^17,19^; this dose was shown to be effective in stimulating regenerative responses in numerous rodent studies spanning a variety of central and peripheral lesion models^47^.

At the conclusion of the study, animals were euthanized, fresh brains were rapidly removed, and two to three striatal samples from both sides were dissected and immediately frozen for later analyses. The remainder of the brain (including the striatum beginning just rostral to the level of the decussation of the anterior commissure) was submersion fixed in 4% paraformaldehyde.

### Behavioral testing

Forelimb use during explorative activity was analyzed using the cylinder test. The test apparatus consisted of a clear Plexiglass cylinder of approximately 33 cm diameter and 50 cm height placed in a dimly lit room in front of a mirror in order to visualize limb use from all angles. Each session was videotaped for later analysis by an observer blind to the animal’s experimental group. The paw contralateral to the side of the AAV-A53T-α-synuclein injection was marked prior to the start of each test session to definitively determine laterality on videotapes. The test was performed in the late afternoon at the same approximate time for each test session. At each test session, the animal was gently placed in the cylinder and movements were videotaped for 10 minutes. Forelimb use was assessed by scoring weight-bearing contacts on the cylinder wall with the ipsilateral, contralateral (relative to the AAV-A53T-α-synuclein-injected side), and both paws. Twenty observations of the paw placements on the cylinder wall were scored.

Limb placement on the wall was scored only if it occurred after the animal returned to a resting position and reared again to touch the cylinder wall. Percent ipsilateral (ipsi) and contralateral (contra) paw touches were calculated as [(ipsi + 1/2 both)/total # observations] * 100 and [(contra + 1/2 both) /total # observations] * 100, respectively. The cylinder test was performed prior to surgery (baseline) and at 3 and 6 weeks after surgery for the immediate start GM1 groups and at 3 and 8 weeks after surgery for the delayed start GM1 groups.

### Measurement of striatal dopamine and metabolite levels

Striatal samples were sonicated cold in 0.4 M perchloric acid for 10 seconds and centrifuged at 13,000 rpm for 10 min at 4 °C. The supernatant was removed and was diluted 1:4 with Milli-Q ultrapure water (resistivity 18.2 MΩ·cm) containing internal standard (isoproterenol, 4 ng). The diluted samples were centrifuged again at 13,000 rpm for 5 min at 4 °C. Samples were kept on ice prior to loading into the refrigerated (4 °C) autosampler integrated with the HPLC system.

An ALEXYS UHPLC system (Antec Scientific) with electrochemical detection (Decade Elite electrochemical detector with a glassy carbon electrode) was used for the analyses. Separations were achieved using an Aquity UPLC column 1.0 × 100 mm BEH C_18_ 1.7 um (Waters Corporation). The mobile phase (pH 3.0) consisted of 100 mM phosphoric acid, 100 mM citric acid, 0.1 mM EDTA.Na_2_, 600 mg/L octanesulfonic acid sodium salt, and 8% acetonitrile. Pump flow rate was 50 µl/min. The analytes were detected at an oxidation potential of 600 mV against a reference electrode at 0 mV. Data were acquired and processed using Clarity software (v7.4.1) (DataApex). Peak heights were compared against standard curves to determine the concentrations of DA and metabolites in each sample.

### Immunohistochemistry and stereological cell counting

Fixed tissue blocks were immersed in 30% sucrose for cryoprotection and sectioned frozen on a sliding microtome (30 μm section thickness for striatal sections; 40 μm section thickness for SN sections). Sections through the rostro-caudal extent of the SN were collected and every sixth section from the SN was processed for tyrosine hydroxylase (TH) immunohistochemistry to be used for stereological cell counting. Three striatal sections from approximately the same anatomical level in each case were processed for α-synuclein immunohistochemistry.

For immunohistochemistry, sections were washed and permeabilized with PBS containing 0.2% Triton X-100. Endogenous peroxidase activity was quenched using 3% hydrogen peroxide in PBS containing 0.2% Triton X-100. Sections were then blocked in PBS/triton X-100 containing 10% normal goat serum and 2% BSA, followed by primary antibody incubation (TH (rabbit anti-TH, 1:2,000, Pel-Freez); α-synuclein (mouse anti-α-synuclein clone 211; 1:2000, Millipore) overnight at 4 °C. The following day, sections were washed in PBS and incubated in biotinylated secondary antibody (goat-anti-rabbit 1:4000 for TH; goat-anti-mouse 1:2000 for α-synuclein 1:2000, VECTOR Laboratories), followed by additional washes and incubation in avidin biotin complex (VECTASTAIN Elite ABC system, VECTOR Laboratories). The reaction product was visualized with 3,3′-diaminobenzidine (DAB) (Immpact DAB, VECTOR Laboratories). Sections were then mounted, dehydrated, cleared and coverslipped. Sections stained for visualization of TH and to be used for stereological cell counting were counterstained with cresyl violet^48^ prior to coverslipping.

Stereological estimates of the numbers of TH and cresyl violet-stained neurons in the SNc on both sides of the brain were obtained using StereoInvestigator software (MBF Bioscience) and an Olympus BX-60 microscope equipped with a Ludl motorized stage. Slides were coded and analyzed blindly. The SNc was outlined under low magnification (4x) and a grid measuring 195 μm × 85 μm was randomly placed over the region. Cells were then counted at high power (100x) using a counting frame measuring 60 μm^2^. A cell was counted only if a nucleus was clearly identifiable and the cell was within the counting frame and did not contact the left or bottom boundary of the counting frame. This process was repeated for each section in the series for a given animal, and a total of 7–8 sections/animal were analyzed. Nissl-stained cells were counted concurrently in the same sections using the same sampling parameters. Cell counts were considered acceptable with a Gundersen CE < 0.1.

### Analysis of α-synuclein and α-synuclein-positive aggregates

Immunoblotting for α-synuclein expression was performed using the Wes western blot system (ProteinSimple). Tissue lysates were prepared in standard RIPA buffer supplemented with protease inhibitors and were resolved using the 12–230 KDa Wes separation module according to the manufacturer’s recommendations. α-synuclein levels were assayed using anti-α-synuclein antibody (Syn204) (Cat. No. 2647S, Cell Signaling) and normalized to β-actin levels (anti-beta-actin antibody, NB600-503, Novus Biologicals) (See Supplementary Material for addition immunoblotting method details).

Recent *in vitro* studies suggest that GM1 and α-synuclein interact in a way that protects monomeric α-synuclein from potentially pathogenic aggregation^32^. We investigated the extent to which GM1 treatment *in vivo* might affect the aggregation of α-synuclein by measuring the size of α-synuclein-positive aggregates in the striatum of AAV-A53T animals treated with saline or GM1. The sizes of α-synuclein-positive swelling/aggregates were measured in 3 fields (one medial, one central and one lateral) from 1 coronal section (at the same approximate anatomical level in each animal). Photographs were captured using a 40x objective on a Nikon Eclipse *Ni* microscope and α-synuclein-positive structures with an area >5 µm^2^ were considered as aggregates and were measured using Nikon NIS Elements AR software. Data are presented as the distribution of aggregate sizes in the three striatal regions measured.

### Exclusion of animals from analysis

A total of nine animals from all surgery groups were excluded from analysis due to no evidence of a successful lesion produced by AAV1/2-A53T α-synuclein injection. For technical reasons or for lack of sufficient tissue available for processing, two animals from the early start A-53T-α-synuclein/saline group and four animals from the early start A-53T-α-synuclein/GM1 group were excluded from analysis of stereological cell counts. Also, for technical reasons or for lack of sufficient tissue available for processing, one animal from the delayed start A-53T-α-synuclein/saline group was excluded from the cylinder test analysis and from the analysis of DOPAC levels; two animals from the delayed start A-53T-α-synuclein/GM1 group were excluded from analysis of stereological cell counts and from analysis of DOPAC levels.

### Statistical analyses

All statistics were performed using GraphPad Prism software (v7). Values are presented as means ± SEM. Comparisons between experimental groups were performed using Student’s t test or when comparisons involved more than two groups, one-way analysis of variance followed by post hoc comparisons using Bonferroni’s multiple comparison test. All analyses were two-sided. The nonparametric Kolmogorov Smirnov test was used to test whether samples of α-synuclein aggregate sizes from saline and GM1-treated animals came from the same distribution or not (i.e., H_0_: the two samples come from a common distribution; H_a_: the two samples do not come from a common distribution). In all cases, statistical significance was set at P < 0.05. All data are available upon request.

## Supplementary information

Supplementary Material

## Data Availability

The datasets generated during and/or analyzed during the current study are available from the corresponding author on reasonable request.
