# Supplementary material for: GM1 Ganglioside Modifies α-Synuclein Toxicity and is Neuroprotective in a Rat α-Synuclein Model of Parkinson’s Disease
Source: Sci Rep. 2019 Jun 10;9:8362. doi: 10.1038/s41598-019-42847-x (PMC6557812; doi:10.1038/s41598-019-42847-x)
Supplement: Supplementary file 1 — Supplementary Material [file 41598_2019_42847_MOESM1_ESM.pdf]

Supplementary Material

GM1 Ganglioside Modifies α-Synuclein Toxicity and is Neuroprotective in a Rat α-Synuclein Model of Parkinson's Disease.

Jay S. Schneider*, Radha Aras, Courtney K. Williams, James B. Koprich[1], Jonathan M. Brotchie[1], Vikrant Singh

Department of Pathology, Anatomy and Cell Biology, Thomas Jefferson University, Philadelphia, PA 19107 and [1]Toronto Western Research Institute, Toronto Western Hospital, University Health Network, Toronto, Ontario, Canada M5T 2S8.

Supplementary Methods: Immunoblotting

Immunoblotting for analyzing α-synuclein and β-actin expression was performed using the Wes automated Western blot system based on capillary electrophoresis and immunodetection (ProteinSimple) for enhanced sensitivity and reproducibility at low sample concentrations. Tissue lysates were prepared in RIPA buffer supplemented with protease inhibitors (Halt Protease and Phosphatase Inhibitor Cocktail, Thermo Fisher Scientific) and quantified using the Micro BCA Protein Assay Kit (Thermo Fisher Scientific). For each sample, sufficient volume of mastermix was prepared for loading two capillary wells enabling assay of target (α-synuclein) and control (β-actin) signals, from equal sample amounts, in individual capillaries to minimize any interference in chemiluminescence signal generated by target and control proteins. Sample lysate mastermix was prepared by combining tissue sample lysate (4μg per well), 0.1X sample buffer and 5X fluorescent mix (200 mM DTT, 5X sample buffer, 5X fluorescent standards) and denaturing at 70°C for 10 minutes. For each sample, equal amount of denatured lysate mastermix was loaded in two capillary wells of a 12-230 KDa Wes separation module plate and resolved according to the manufacturer's recommendations. α-synuclein levels were assayed using anti-α-synuclein antibody (Syn204) diluted 1:25 (Cat. No. 2647S, Cell Signaling) and β-actin levels were assayed using anti-beta-actin antibody diluted 1:50 (NB600-503, Novus Biologicals). HRP conjugated anti-mouse (Cat no. 042-205) and anti-rabbit secondary antibodies and luminol detection reagents (Cat no. DM-001) were obtained from ProteinSimple. The expression levels of α-synuclein and β-actin were determined by calculating the area under the curve for α-synuclein and β-actin chemiluminescence signals using the Gaussian distribution function of Compass Software (Protein Simple). Finally, α-synuclein signal was normalized to β-actin signal and presented. The Wes Western Immunoblot images were prepared using Compass Software (ProteinSimple).

Supplementary Table S1. Effects of AAV-A53T-a-synuclein and GM1 ganglioside administration on striatal dopamine (DA) and dihydroxyphenylacetic acid (DOPAC) levels.

Treatment	*n*	DA (µg/g wet tissue)	DOPAC (µg/g wet tissue)
AAV-A53T/Saline: Non-Injected Side (6 wks)	15	10.34 ± 0.47	1.02 ± 0.08
AAV-A53T/Saline: Injected Side (6 wks)	15	4.99 ± 0.55	0.79 ± 0.09
AAV-A53T/Early GM1: Non-Injected Side	21	11.43 ± 0.32	1.33 ± 0.10
AAV-A53T/Early GM1: Injected Side	21	7.29 ± 0.45[a]	0.85 ± 0.09
AAV-A53T/Saline: Non-Injected Side (8 wks)	12	11.44 ± 0.59	1.43 ± 0.18^
AAV-A53T/Saline: Injected Side (8 wks)	12	3.62 ± 0.54	0.85 ± 0.09^
AAV-A53T/Delayed GM1: Non-Injected Side	19	10.73 ± 0.54	1.18 ± 0.10^
AAV-A53T/Delayed GM1: Injected Side	19	5.87 ± 0.47[b]	0.93 ± 0.12^

[a]P = 0.0026 vs. AAV-A53T/Saline; [b]P = 0.004 vs. AAV-A53T/Saline; ^Two samples were determined to be extreme outliers by Grubbs' test and were removed from the analysis.

Supplementary Table S2. Effects of AAV-A53T-a-synuclein and GM1 ganglioside administration on substantia nigra pars compacta dopamine neurons.

Treatment	*n*	TH^+ Cells	$Nissl^+$ Cells
AAV-A53T/Saline: Non-Injected Side (6 wks)	13	5831 ± 189	6174 ± 157
AAV-A53T/Saline: Injected Side (6 wks)	13	2314 ± 140	2784 ± 128
AAV-A53T/Early GM1: Non-Injected Side	17	5601 ± 135	6074 ± 174
AAV-A53T/Early GM1: Injected Side	17	3144 ± 159[a]	3557 ± 172[b]
AAV-A53T/Saline: Non-Injected Side (8 wks)	12	5621 ± 183	5972 ± 172
AAV-A53T/Saline: Injected Side (8 wks)	12	2052 ± 177	2480 ± 159
AAV-A53T/Delayed GM1: Non-Injected Side	17	5685 ± 143	6113 ± 132
AAV-A53T/Delayed GM1: Injected Side	17	2814 ± 134[c]	3335 ± 148[d]

[a]P = 0.0008 vs. AAV-A53T/Saline; [b]P = 0.002 vs. AAV-A53T/Saline; [c]P = 0.0017 vs. AAV-A53T/Saline; [d]P = 0.0006 vs. AAV-A53T/Saline.

Supplementary Figure 1.

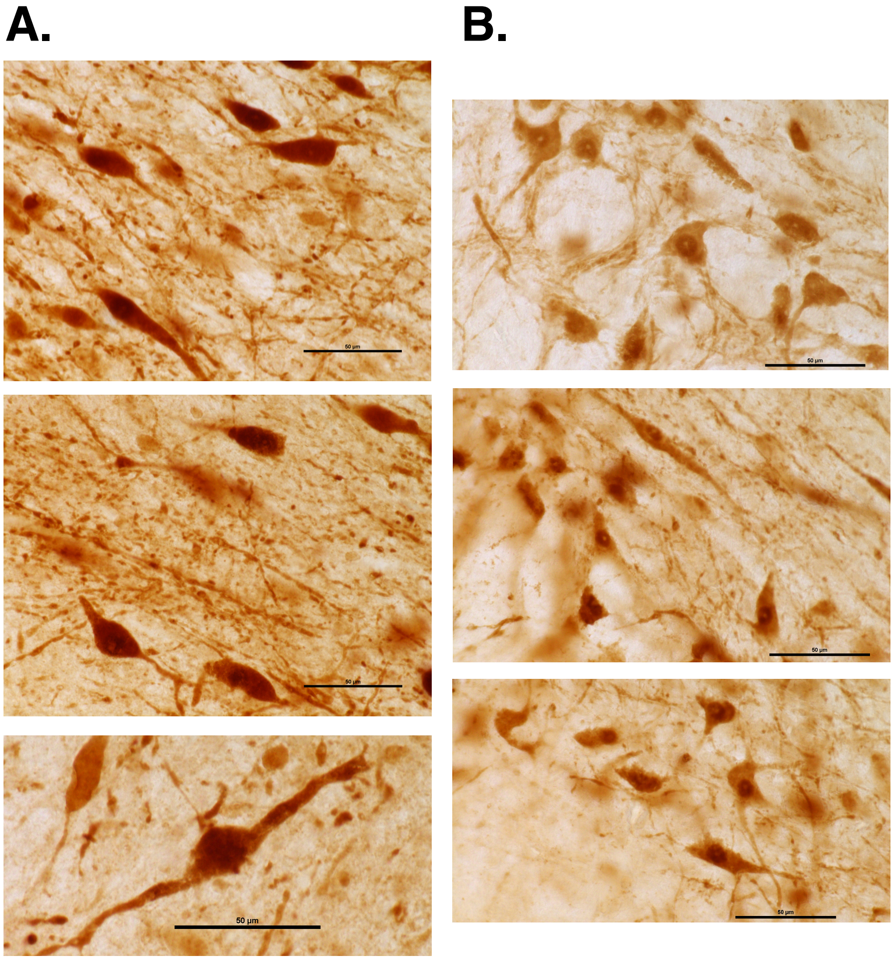

Supplementary Figure 1. Immunohistochemical staining of SN sections for visualization of Ser129 phosphorylated α-synuclein in saline-treated (A) animals 8 weeks after AAV-A53T- α-synuclein injection and delayed start GM1-treated animals (B). Ser129 phosphorylated α-synuclein staining appeared more intense and appear to fill more of the neuron in saline-treated animals compared to GM1-treated animals.

Supplementary Figure 2.

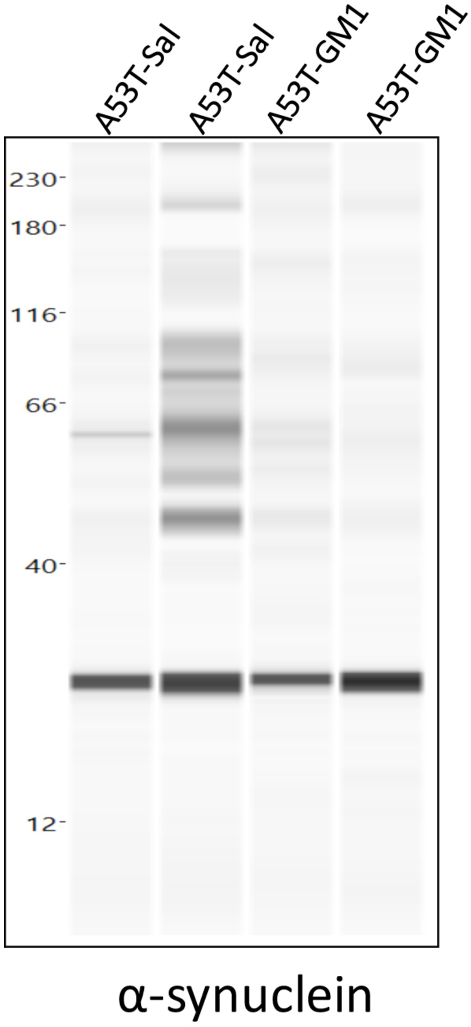

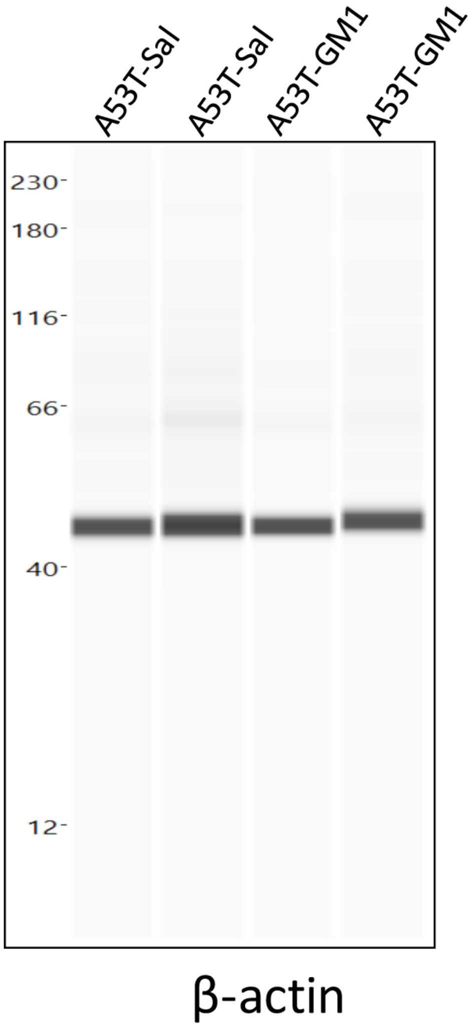

Supplementary Figure 2. Full length Wes immunoblots of images presented in Figure 2.
